# Molecular marker assisted gene stacking for disease resistance and quality genes in the dwarf mutant of an elite common wheat cultivar Xiaoyan22

**DOI:** 10.1186/s12863-020-00854-2

**Published:** 2020-04-23

**Authors:** Weijun Zheng, Song Li, ZiHui Liu, Qi Zhou, Yanru Feng, Shoucheng Chai

**Affiliations:** State Key Laboratory of Crop Stress Biology for Arid Areas, College of Agronomy, Northwest A&F University, Yangling, 712100 Shaanxi China

**Keywords:** Wheat, Marker-assisted selection, Powdery mildew resistance, Yellow rust resistance, Grain quality.

## Abstract

**Background:**

Development of wheat cultivars with multiple disease resistance and high quality are major objectives in modern wheat breeding programs. Gene stacking is an efficient approach to achieve this target. In this study, we pyramided yellow rust resistance gene (*Yr26*), powdery mildew resistance gene (*ML91260*) and high-molecular-weight glutenin subunits *Dx5 + Dy10* into the dwarf mutant of an elite wheat cultivar, Xiaoyan22.

**Results:**

Six pyramided wheat lines were obtained by molecular marker-assisted selection (MAS) and field evaluation of disease resistance. The desirable agronomic traits of pyramided lines, their identity with the original cultivar Xiaoyan22 except for plant height, tiller number and disease resistance, was achieved in this study. Meanwhile, the yield of pyramided lines is higher than Xiaoyan22 in the field test. In addition, analysis of flour quality indicated that the dough stability time of pyramided lines was longer than that of Xiaoyan22.

**Conclusions:**

Six pyramided wheat lines with two disease resistance and high quality were achieved in this study. It is feasible to improve multiple agronomic traits simultaneously by rational application of MAS.

## Background

Wheat (*Triticum aestivum*L.)is a staple food crop that feeds more than 35% of the population in the world, contributing nearly 20% of the total calorie intake [1, 2]. It is cultivated mainly in Europe and Asia, and used for chapati, biscuit, bread and noodles. China is the largest wheat production country, producing more than 1.3 hundred million tons in 2017 [3]. The Huang-Huai plain is the major region for wheat production in China accounts for 69.2% of the wheat yield [4]. However, wheat powdery mildew and yellow rust are becoming the primary adverse factors that limit wheat yield in this area. Wheat powdery mildew (PM, caused by *Blumeriagraminis* (DC.) Speer*, Erysiphe graminis*D.cf.sp*.tritici*E. Marchal*)*(*Bgt*) and yellow rust (YR caused by *Puccinia striiformis*West. f.sp.*tritici*Eriks et Henn)(*Pst*)*,* are two major fungal diseases. Those two pathogens can attack all above-ground organs of wheat, including leaves, stems, and spikes, cause significant losses in wheat production [5, 6]. Since 2000, the occurrence of wheat PM disease was maintained at 600 × 10^4^ hm^2^ [7] and it caused annual yield losses of between 10 and 20% of the total harvest as reported by Cao [8]. Meanwhile, the wheat YR has become the largest biotic limitation to wheat production in the Huang-Huai area and drastically threatens food supply. Thus, there is an urgent need to use more effective PM and YR resistance genes or pyramiding different resistance genes to develop more durable resistant wheat cultivars in Huang-Huai area as well as other wheat-producing regions.

On the other hand, the wheat quality is becoming a concern of the consumer. The alleles of the high-molecular-weight glutenin subunit gene determines the processing quality of wheat in most cases. HMW glutenins are encoded by the *Glu-1* loci on the long arms of chromosomes 1A, 1B and 1D, and these loci are designated *GluA1*, *Glu-B1* and *Glu-D1*, respectively. Each locus consists of two tightly linked HMW glutenin genes, one x-type and one *y-*type. The glutenin subunits encoded by 5 + 10 alleles of *Glu-D1*are important for determining dough properties, SDS-sedimentation, and loaf volume [9]. At present, the main cultivated varieties of wheat in Huang-Huai are medium-gluten wheat, and the wheat cultivars with strong gluten are still deficient. This indicates that wheat quality must be improved along with improving PM and YR resistance. Improving multiple agronomic traits simultaneously is a difficult task. Owing to the advance of the marker-assisted selection (MAS) technique, it is feasible to stack multiple genes into one cultivar [10, 11]. In common wheat, eight QTLs/genes for seven different traits were pyramided into a variety PBW343, and the new pyramiding lines (PYLs) were resistant against three rust pathogens, and the grain quality was improved [12]. In soybean, the genes *Rsv1, Rsv3,* and *Rsv4* which confer resistance to seven strains of soybean mosaic virus (SMV) were introgressed into soybean by MAS and generated a variety that is resistant to SMV [13]. In rice, bacterial blight resistance genes *Xa13* and *Xa21*, were introgressed into *indica* rice cultivar PR106 also by MAS; those two genes provided durable blight resistance to Indian Rice Variety MTU1010 [14].

Xiaoyan22(XY22) is medium-gluten flour wheat variety, obtained from cross breeding of XiaoYan6/775–1//XiaoYan107 by Northwest A&F University, China [15]. Since this variety was approved in 1998, it has been planted in 26.7× 10^4^to 40.0 × 10^4^ hm^2^ in Huang-Huai areaand central Shaanxi provinceevery year [16].XY22 is noted for producing high yields under adverse abiotic and biotic stress. After standing as the yield control in Shaanxi province for 29 years, XY22 is losing the resistance to YR and PM. Meanwhile, plant height is too high (about 80 cm) and thus susceptible to lodging, and poor grain quality has also emerged as a concern by farmers in recent years. In this study, an attempt to simultaneously improve the above four defects of XY22 were conducted by MAS and field evaluation of disease resistance. *Ml92160–1* and *Ml91260–2,*two complementary genes*,*determine resistance to *Bgt*isoloateE09 at the adult stage [17].*Yr26,*an YR resistance gene*,* is effective against the *Pst* race CYR32 and CYR33 [18].Yr26*,Ml92160–1* and *Ml91260–2,*along with *Glu-D1–5* and *Glu-D1–10,*two wheat flour quality related genes, are selected as the target for pyramiding. Six pyramided lines (PYLs) with short height, resistance to YR and PM, and high grain quality, were obtained. The yield of those PYLs are higher than the parent cultivar XY22. These PYLs with high disease resistance and high grain quality are likely to be used in the future as germplasm resources in wheat breeding and perhaps even as new wheat cultivars.

## Methods

### Plant materials and breeding strategy

Xiaoyan22 was acquired from Researcher Zhang Li, Northwest A&F University, China.Xiaoyan22D with shorter plant height, which was discovered by ourself in population XY22, was a spontaneous mutant, and served as the recipient parent for improving lodging resistance of XY22 (Fig. S1). The other three donors as follows: (l) 91,260 with two dominant complementary genes *ML91260–1* and *Ml91260–2* conferring resistance to *B. graminis* f. sp. *Tritici*was a gift from the Dr. Zhen Fang, Agricultural Research Center of Shaanxi [17] . (2):92R137 with gene *Yr26* conferring resistance to *P. striiformis*West. f.sp.*tritici*was got from Dr. Peidu Chen, Nanjing Agricultural University [19]. (3) ZhengNong 16 with two tightly linked *Glu-D1* genes encoding high-molecular-weightglutenin subunits (HMW-GS), *Glu-D1d*(Dx5 + Dy10) was developed by Tiwen Lei, Academy of agriculture and forestry of Zhengzhou [20] .These four parents were crossed in pairs to produce three single-cross F1 hybrids, then intercrossed to produce a double-cross F1 hybrid (DCHF1). “The genotype Ml91260-1, Ml91260-2, Yr26” of DCHF1 plants was verified with applying molecular marker analysis and field evaluation of resistance.” The verified DCHF1 with genotype (*Ml91260–1, Ml91260–2, Yr26*) was crossed with the third single-cross F_1_ hybrids produce a three-cross F1 hybrid (TCHF1) for pyramiding of disease resistance genes and HMW-GS *Dx5 + Dy10*. The plants of TCHF1 with genotype (*Ml91260–1, Ml91260–2, Yr26, Dx5 + Dy10)* were backcrossed with XY22D two times to produce plants which agronomic traits similar to those of XY22D (TCHF1BC1 to TCHF1BC2) with MAS and field disease resistance evaluation. The subsequent two generations (TCHF1BC2F1 to TCHF1BC2F2) were raised through selfing, with MAS exercised and agronomic traits investigated in each generation, eventually leading to selection of six improved groups, including 18 lines, designated Pyramided lines (PYLs) and Plants (PYL1–6; Plant 1–18, Fig. S1). The 18 plants were multiplied individually to produce sufficient progenies for evaluation of phenotypic traits, yield and grain quality.

### DNA extraction and PCR amplification

In each generation, marker assisted selection (MAS) was exercised using simple sequence repeat (SSR) markers for tracking specific genes. The totalDNA for polymerase chain reaction (PCR) amplification of every generation of plant material was extracted according to the procedure ofSharpPJ et al. [21]. Specific SSR primerswhich are adjacent to *ML91260–1, ML92160–2, Yr26* and *Dx5 + Dy10* [17, 19, 22], were adapted for PCR amplification (Table 1)*.* Total reaction mixture was 15 μL containing: 100 ng of genomic DNA, 1X Taq DNA polymerase buffer, 10 pmol of forward and reverse primers, 2.5 mM of each dNTP and 0.15 U of Taq DNA polymerase (Takara, USA). Template DNA was initially denatured at 95 °C for 5 min, prior to 32 cycles of denaturation at 94 °C for 1 min, annealing at 50–65 °C for 45 s and extension at 72 °C for 30 s. In the final step, the reaction mixture was incubated at 72 °C for 10 min before completion. After amplification, 3 μL of restriction buffer (10×) was added to the PCR products, which were separated on a 4% agarose gel.

**Table 1:** Traits, related genes, their chromosomal location and linked molecular markers [with their product size in donor(D) and recipient (R) genotypes] used for marker-assisted selection

Trait	chromosome	Linked marker	Genetic distance between marker and gene	Product size (bp)	Reference
yellow rust	1BS	*Xgwm18*	1.9 cM	182(D), 188(R)	Ma J et al. (2001)
5 + 10 subunit	1D	*Dx-F, Dx5-F, Dx-R*	0 cM	320 bp, 343 bp, 361 bp	Ishikawa et al. (2007)
powdery mildew	2AL	*Xwmc170*	4.1 cM	257(D), 248(R)	Zhang et al. (2015)
powdery mildew	2BL	*Xwmc332*	6.7 cM	228(D), 236(R)	Zhang et al. (2015)

### Sodium dodecyl sulfate-polyacrylamide gel electrophoresis (SDS-PAGE)

SDS-PAGE for analysis of HMW glutenin subunits *Dx5 + Dy10* in the TCHF1BC2F3 was conducted as described by Damaniawithminor modifications, the separating gel concentration is 12% and thestacking glue is 8% [23]. Proteinwere extracted from half seeds of each plant and electrophoresis was performed at a constant voltage of 200 V with a low concentration of running buffer [16.7 mM Tris 127.9 mM glycine, 0.07% (w/v) SDS]. Electrophoresis was continued until protein bands carrying low molecular weightcomponents reached near the end of the gel.

### Evaluation of agronomic traits and disease resistance of XY22 and the PYLs

Six PYLs and control XY22 were sown at the experiment station of Northwest A&F University in ShaanXi province with three replications from 2015 to 2017.Each entry consisted of eight rows (4 m) with 161 seeds per row and a row-to-row distance of 25 cm. Seeds of XiNong2000, a variety highly susceptible to yellow rust and powdery mildew were sown at the border as a guard row. Leaves of plants in the guard row were inoculated with *Pst*isolate E09 and *Egt*isolates TiaoZhong33 at shooting period. Inoculations were performed by brushing condidiafrom neighboring sporulating susceptible plants of XiNong2000 from booting stage to heading stage. E09 pathotypes caused severepowdery mildewepidemics in the wheat-growing regions of north China [8] and TiaoZhong33 pathotypes caused severe yellow rust epidemics in the wheat-growing regions of south GanSu and central ShaanXi, China [24]. Data were recorded on the following nine traits: (1) plant height at shooting period and maturation period (cm), (2) powdery mildewresistance, (3) yellow rust resistance, (4) grain quality, (5) kernel number of 30 spikes, (6) 1000-grain weight (g),(7) grain yield (t/ha), (8) productive tillers in 1m^2^,(9) days to heading and flowering. The PM severity was scored using the 0–9 scale as descried by A Blancoet al [25]. Plants with scores 0–4 were classified as resistant,and withscores 5–9 as susceptible. The disease symptomswere scoredusing the 0–4 scale according to resistance level as described by RAMcIntoshet al [26] for YR.Scores0–2 indicated resistance, scores 3–4 indicated susceptibility. A strong wind, resulting in the lodging of XY22, occurred in May of 2017. Thus, the data was investigated only in 2015 and 2016.

### Flour quality analysis of XY22 and PYLs

For each PYL, 1.5 kg was analyzed for flour quality. Wheat gluten content was analyzed by Glutograph-E (Germany). Wheat bulk density was measured with a bulk density meter (BLH-5000, China). Dough formation time and stability timewere analyzed by a wheat extensograph (Farinograph-E, Germany).

### DATA analysis

Comparison of the means of the data of all nine agronomic traits in 2 years were performed using the analysis of variance. The data on grain yield (kg) was converted into grain yield (t/ha) for further statistical analyses. *P* values less than 0.05 were considered to be statistically significant. Statistical analysis was performed using the SPSS 19.0 software (IBM Inc. Armonk, NY, USA).

## Results

### PyramidingPM/YR resistance genes and HWM-GS by MAS

**Fig. 1:**
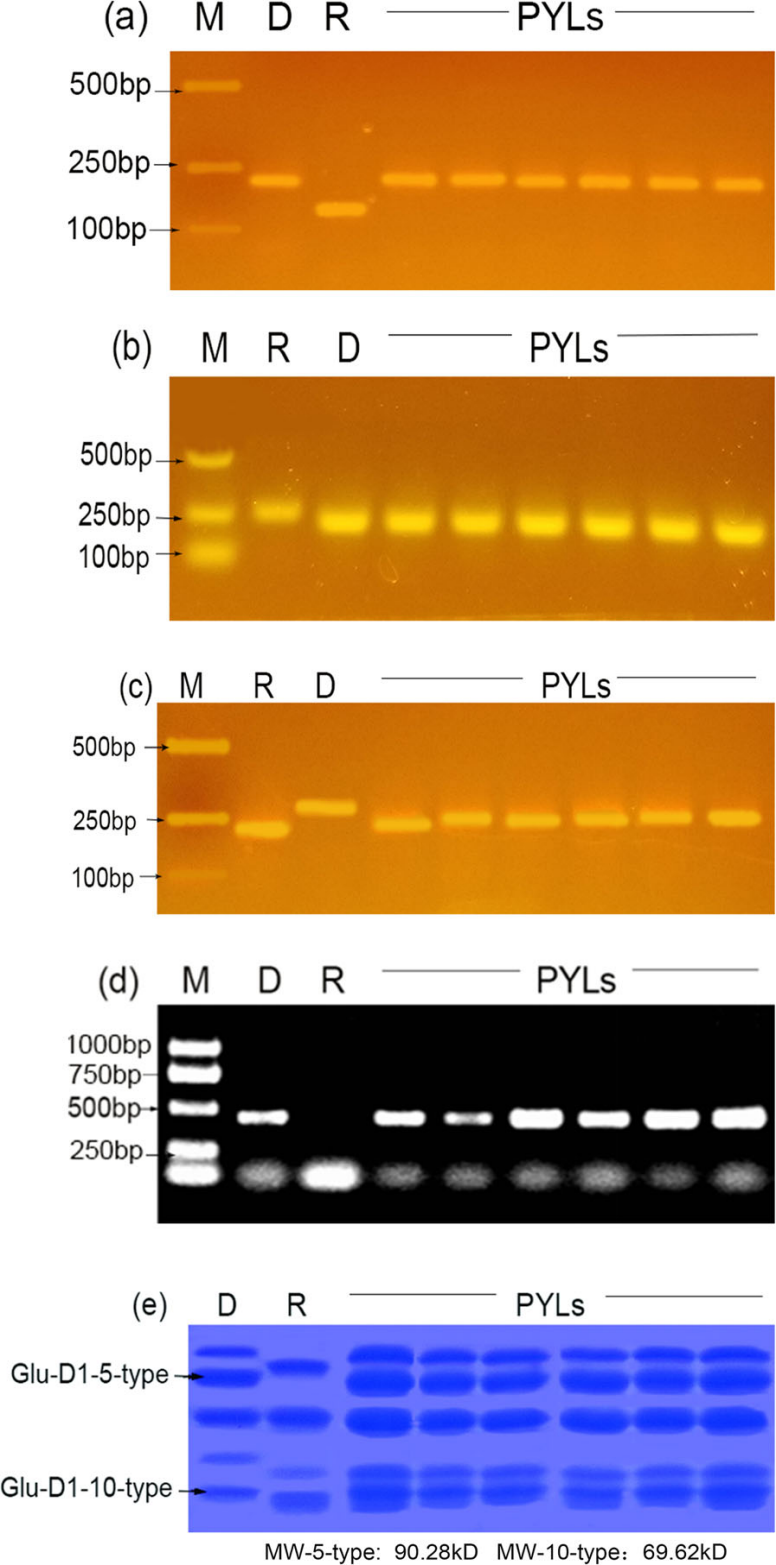
Molecular detection of resistance genes *Yr26, Ml91260* and HWM-GS, forsomePYLs derived from TCHF1BC2F2, and their parents. **a**: yellow rust (*Xgwm18*); **b** and **c**: powdery mildew (*Xwmc170*) and (*Xwmc332*); **d**:5 + 10 dominant markers; **e**: Electropherogram of grain HMW-GS, MW: molecular weight. M: DNA molecular weight marker DL2000; D: donor parent; R: recipient parent; PLYs:pyramided lines, the same below

With appropriate DNA markers for yellow rust and powdery mildew resistance and HWM-GSDx5 + Dy10, marker selection was exercised in each generation from DCHF1 to TCHF1BC2F2 (Table S1). *Ml91260–1, Ml91260–2, Yr26* or *Dx5 + Dy10* genes were detected in each generation. Foreground selection was exercised in TCHF1BC1 to TCHF1BC2 to select plants that carried all genes in heterozygous condition; 11 plants were selected and used to raise TCHF1BC2F1. Screening of TCHF1BC2F1 with co-dominant markers (*Xcfwmc170, Xwmc332, Xgwm18* and *Dx-F, Dx5-F, Dx-R*) linked to *Ml91260–1, Ml91260–2, Yr26* and *Dx5 + Dy10*, resulted in the selection of nine plants of 233 progenies, which were homozygous for three resistance genes but heterozygous for HWM-GS Dx5 + Dy10. Based on MAS and SDS-PAGE, six plants from TCHF1BC2F2 were found to be homozygous for all targeted genes. Those six plants were then selfed to producePYL 1–6. The DNA of all pyramided lines, recipient parent and donor parent were analyzed for resistance genes and *Dx5 + Dy10*. The results indicated that all pyramided lines contained three resistance genes (Fig. 1 a, 1 b,1 c) and HWM-GS *Dx5 + Dy10* (Fig. 1 d), but the recipient parent did not. Proteins of wheat grain of the PLYs, recipient parent and donor parent were extracted and detected by SDS-PAGE. As expected, all PLYs contained the 5 + 10 subunits, but XY22 did not (Fig. 1 e).

### Evaluation of YR resistance and PM resistance

**Fig. 2:**
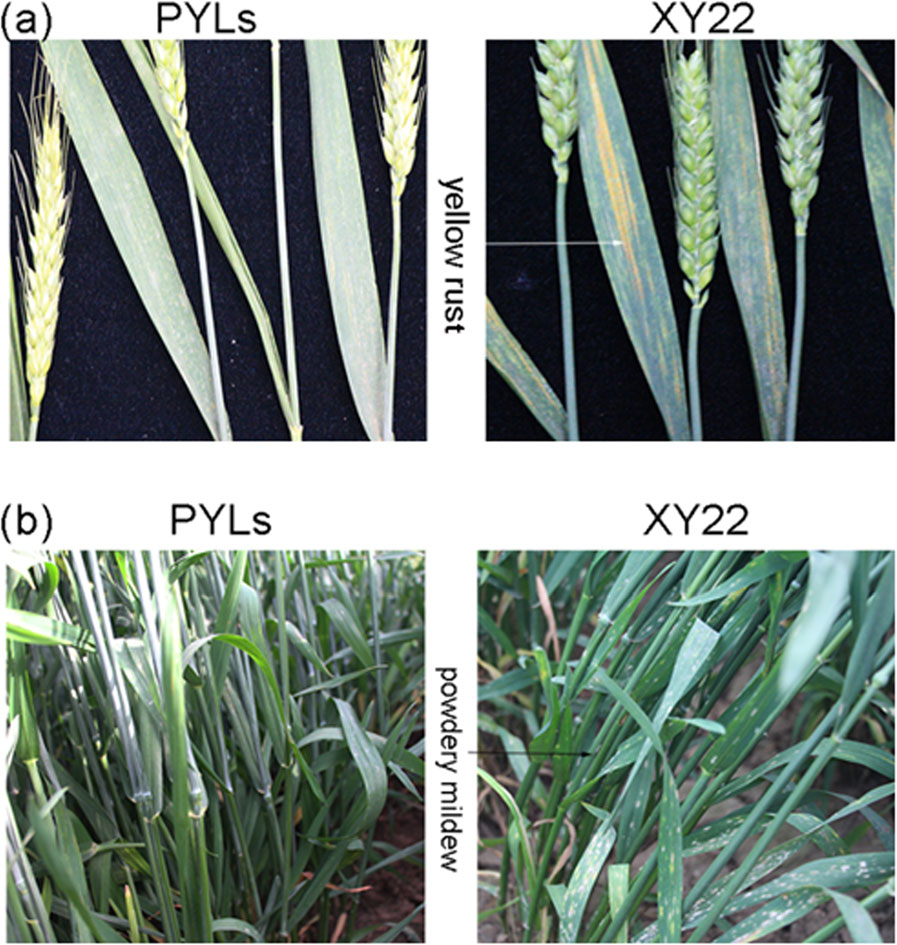
Two disease resistances performance of PLYs and XY22. **a**: yellow rust; **b**: powdery mildew

The evaluation of YR resistance and PM resistance were carried out along with the yield test from 2015 to 2017. The resistance of PYLs and XY22 wasconstant in evaluations in the 3 years. As expected, the resistance score of YR of PLYs were 1–3, compared to 4 for XY22 (Fig. 2 a, Table 2). Notably, PLY 4 and PLY 5 were immune to the YR pathogen. Evaluations of the PLYs for PM ranged from highly resistant to moderately resistant versusmoderate susceptibility for XY22 (Fig. 2 b, Table 2). Disease resistance in all pyramided lines was superior to that of XY22.

**Table 2:** Means and standard deviations (SD) of 10 phenotypic traits of the recipient cultivar and six pyramided lines (PYLs)

Traits	Lines						
	XY22	PYL1	PYL2	PYL3	PYL4	PYL5	PYL6
Plant height (cm) -Shooting period	36.83 ± 2.5	41.57 ± 2.4*	40.57 ± 1.7*	42.00 ± 2.1*	42.67 ± 2.7*	40.53 ± 1.6*	46.47 ± 2.2*
Plant height (cm)-Maturation period	72.43 ± 2.4	67.00 ± 2.0*	64.80 ± 2.7*	65.57 ± 2.2*	65.87 ± 2.4*	67.2 ± 1.7*	66.77 ± 2.5*
Days to flowering	199 ± 0.25	198 ± 0.25	197 ± 0.25	197 ± 0.25	198 ± 0.25	198 ± 0.25	197 ± 0.25
yellow rust resistance	4	2*	3*	2*	1*	1*	2*
Powdery mildew resistance	5	3*	2*	3*	2*	2*	2*
1000 grain weight (g)	41.35 ± 1.52	42.01 ± 1.56	43.4 ± 0.7	41.21 ± 0.86	43 ± 1.2	41.69 ± 0.5	40.3 ± 1.64
Tiller of 1m^2^	449 ± 14	512 ± 12**	492 ± 8**	487 ± 22**	534 ± 13**	566 ± 22**	593 ± 18**
Kernel number of 30 tassels	1220 ± 50	1201 ± 55	1148 ± 60	1127 ± 37	1107 ± 40	1105 ± 27	1237 ± 54
Grain yield (t/ha)	6.6 ± 0.2	7.6 ± 0.3 **	7.2 ± 0.1 **	7.4 ± 0.6 **	7.7 ± 0.3 **	7.5 ± 0.5 **	8.3 ± 0.2 **

variance values are shown in parentheses, * means PYLs with Xy22 have significant difference (*P* < 0.05), **means PYLs with XY22 have highly significant difference

### Evaluation of agronomic traits

**Fig. 3:**
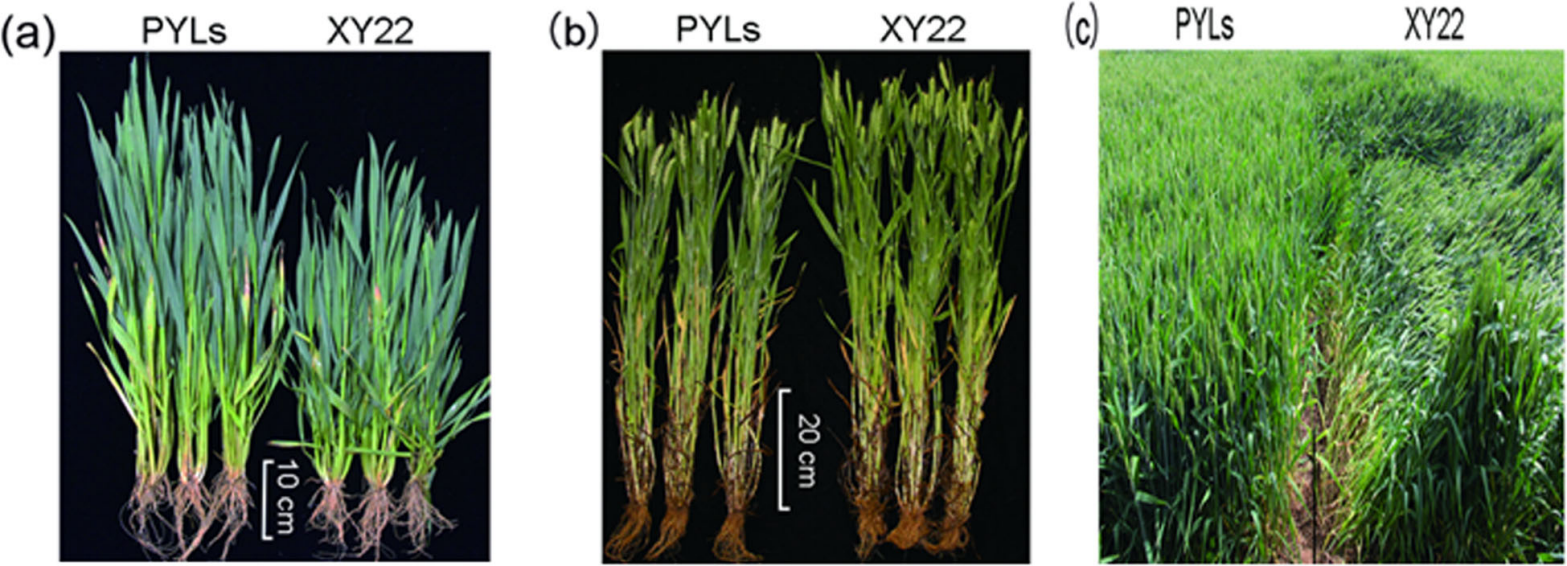
Agronomic traits performance of XY22 and PLYs. **a**: plant height at shooting period; **b**: plant height at maturation period; **c**: lodging resistance comparison

The agronomic traits of XY22 and PLYs were compared in the field. The results showed non-significant differences for plant type and panicle type between XY22 and PLYs (Fig. 3 and 3 b). Plant height was significantly different between XY22 and PLYs at the shooting and maturation periods. The height of PLYs were higher than XY22 at the shooting period (Fig. 3 a). However, the heights of PLYs were less than XY22 at the maturation period (Fig. 3 b). The average height of PYLs was 66.4 cm, which is 6 cm shorter than the mean height of XY22 (Table 2). The stormy weather caused considerable lodging in ShaanXi province in May of 2017. The XY22 was severely lodged at that time, but the PLYswere upright and not affected (Fig. 3 c).

The key components of yield are spike number, grain number per spike, and grain weight. We investigated these three factors in the PLYs and XY22 in 2015 and 2016. The results showed that the mean of 1000-grain weight (g) and kernel number per spike of XY22 and PYLs were not significantly different, whereas the average tillers per 1 m^2^ of PYLs exceeded XY22 by more than 8.0%. In particular, the PYL6 produced the maximum number of tillers among the PYLs, which was 19% greater than XY22. When we converted the yield of experimental plots to yield per hectare, the yield of all PYLs were all higher than XY22. PYL6 produced the highest yield among the PYLs, which was 25.8% greater than XY22. The flowering of PYLs occurs 0.5–2 days earlier than in XY22.

### Evaluation of grain quality

The grain quality and flour quality of PYLs and XY22 were investigated in 2015 and 2016. The mean of the bulk densities of PYLs and XY22 were 753.7 g/L and 751.5 g/L, respectively, with no significant difference between them. The wheat gluten content and wet gluten of PYLs were similar to values for YX22, whereas the dough formation time of PYL1, PYL2 and PYL4 were significantly different from XY22. All of the PYLs had dough stability times significantly longer than XY22. The dough stability time of XY22 ranged from 5.3 min to 6.1 min; the PLYs ranged from 8.1 min to 10.1 min (Table 3).

**Table 3:** Means and standard deviations (SD) of five flour quality traits of PLYs and recipient cultivar

Traits	Lines						
	XY22	PLY1	PLY2	PLY3	PLY4	PLY5	PLY6
wheat bulk density (g/L)	751. ± 12	752 ± 15	743 ± 11	752 ± 9	766 ± 17	754 ± 13	755 ± 15
wheat gluten content (%)	15.1 ± 0.7	15.1 ± 0.6	15.4 ± 1.0	15.3 ± 0.7	14.9 ± 0.6	14.9 ± 0.6	15.0 ± 0.8
wet gluten (%)	31.28 ± 2.3	31.05 ± 2.3	31.82 ± 1.9	31.82 ± 2.1	30.91 ± 2.5	31.1 ± 1.7	32.83 ± 2.7
dough formation time (minutes)	5.3 ± 0.2	5.7 ± 0.3 *	5.5 ± 0.2 *	5.2 ± 0.3	5.8 ± 0.3 *	5.3 ± 0.2	5.1 ± 0.2
dough stability time (minutes)	5.7 ± 0.4	9.7 ± 0.4 **	8.6 ± 0.5 **	8.2 ± 0.3 **	9.0 ± 0.4 **	8.4 ± 0.3 **	9.2 ± 0.4 **

* means PLYs with Xy22 have significant difference (*P* < 0.05), **means PLYs and XY22 have extremely significant difference

## Discussion

Breeding elite wheat cultivars that are developed with resistance to diverse diseases and have good grain quality is a goal commonly pursued by wheat breeders. Multigene pyramiding by MAS provides an efficient method for breeders to improve multi-traits simultaneously. Results of many prominent research programs have demonstrated that multigene pyramiding is feasible in breeding elite crop cultivars [27–29]. However, few reports document the effectiveness of pyramiding resistance genes and quality genes in wheat. In this study, we performed MAS pyramiding of the *Yr26, ML91260* and HWM-GS *Dx5 + Dy10* genes into the dwarf mutants of elite wheat cultivar Xiaoyan22. Our results show that the improved Xiaoyan22 is resistant to YR, PM and lodging and have better grain quality than Xiaoyan22.

### The recurrent parent selection and pyramiding strategy

Selecting the recurrent parent is the first step in multigene pyramiding. Considering the ultimate target for multigene pyramiding is a new variety or cultivar, the elite cultivar of thewheat production is often chosen as the recurrent parent. In this study, we chose XY22, the yield standard in ShannXi province for 29 years, as the recurrent parent. When we further analyzed the defects in XY22, preventing lodging was an additional breeding target for improvement of XY22. Fortunately, we found a natural dwarf mutant (XY22D) within the XY22 population. The agronomic traits of XY22D are common to XY22 except for plant height. Therefore, XY22D was chosen as the recurrent parent. After the recurrent parent has been selected, there are usually two strategies to pyramid multigenes into the recurrent parent. (1) Each target gene is introgressed separately into the recurrent parent and then the recurrent parent containing different genes is crossed with each other; (2) each target gene is introgressed into the recurrent parent one by one. We adopted the first method to pyramid two powdery mildew resistance genes (*ML91260–1,* and *ML91260–2*), one yellow rust resistance gene (*Yr26*) and one HWM-GS gene (*Dx5 + Dy10)* into XY22D. Although this strategy requires a longer time, compared with second strategy, it provides a better opportunity to obtain maximum recurrent parent genome containing the desired genes [12].

### MAS combining disease resistance evaluation in the field to confirm the introgression of resistance genes

The genetic marker is the key to stacking different genes into the receptor plant by MAS. *Xcfwmc170, Xwmc332, Xgwm18* which are linked to *ML91260–1*, *ML91260–2* and *Yr26* [30, 31], respectively, were chosen to stack the powdery mildew and yellow rust resistance genes into XY22D. Considering that *Xcfwmc170, Xwmc332, Xgwm18* are SSR markers*,* the possibility still exists that recombination can occur between markers and genes. This situation will break the linkage between the marker and target genes, which eliminate the function of the marker gene as an indicator. Evaluation of disease resistance in the field was needed to assure that target genes are indeed incorporated into the receptor. In this study, we adopted the strategy of MAS combined with disease resistance testing tocheck the pyramiding lines from DCHF1 to TCHF1BC2F2. This strategy was efficient; six pyramided lines were obtained with improved disease resistance compared to XY22.

### Phenotypic and yield characterization of pyramided lines

The PLYs are significantly shorter than XY22, but the growth period between PLYs and XY22 was not significantly different. The PYLs are more resistant to lodging than XY22 as shown in Fig. 3 c. Furthermore, the yields of the PLYs are higher than XY22. Reducing the plant height can increase the harvest index, perhaps explaining the yield advance in PYLs. However, aadditional characteristic provide major explanations for the yield improvement in PYLs. PYLs produce more tillers than XY22. Spike number, grain number per spike and grain weight are the key components of yield. Although improving any of them will contribute to an increase in yield, the spike number is regarded as the major factor contributing to genetic improvement ofwheat yield [30, 31]. In our study, the PYLs indeed have higher grain production than XY22 by increasing the spike number. The reason for spike number increasing will be investigated in future studies.

### Grain and flour quality characterization of pyramided lines

The wheat storage proteins are the determining factor for the end-use quality. We found no significant difference in protein content between the XY22 and PYLs. We then investigated the flour quality of XY22 and PYLs. The dough formation time and dough stability time of PYLs are longer than those of XY22. AfterPayne scores for quality were formalized in 1983, numerous studies have demonstrated that this system effectively and accurately evaluates the end-use quality of wheat germplasm [32]. The wheat germplasm with the Glu-D1 Dx5 + Dy10 HWM-GS resulted in the highest quality measurement, which is consistent with the Payne scores. Our results confirmed this point. More importantly, our work provides solid evidence that the wheat end-use quality can be improved by manipulating the HWM-GS.

## Conclusion

The multigene pyramiding method is a feasible strategy to develop wheat cultivars with beneficial agronomic traits. In this study, we successfully obtained six pyramided lines with high yield, high disease resistance, and high grain quality by the MAS method. These six pyramided lines can be used as genetic resources in future wheat breeding efforts and may be released as an improved version of XY-22 with superior grain quality and enhanced resistance to diseases.

## Supplementary information

**Additional file 1: Figure S1.** Flow diagram showing various steps involved in pyramiding of genes in wheat XY22D.

**Additional file 2: Table S1.** Steps involved, and foreground selection exercised for population advancement and pyramiding of genes into the genetic background of wheat XY22D.

## Data Availability

All data generated or analysed during this study are included in this published article and its supplementary information files (Additional files 1 and Additional files 2).

## Abbreviations

YR: Yellow rust
PM: Powdery mildew
MAS: Marker assisted selection
SMV: soybean mosaic virus
SSR: Simple sequence repeat
PYL: Pyramided line
XY22: Xiaoyan22;
HMW: High-molecular-weight
DCHF1: Double -cross F1 hybrid
TCHF1: Three-cross F1 hybrid
TCHF1BC1: Backcross generation one of three-cross F1 hybrid
