# Supplementary figures and images for: Molecular marker assisted gene stacking for disease resistance and quality genes in the dwarf mutant of an elite common wheat cultivar Xiaoyan22
Source: BMC Genet. 2020 Apr 23;21:45. doi: 10.1186/s12863-020-00854-2 (PMC7178591; doi:10.1186/s12863-020-00854-2)

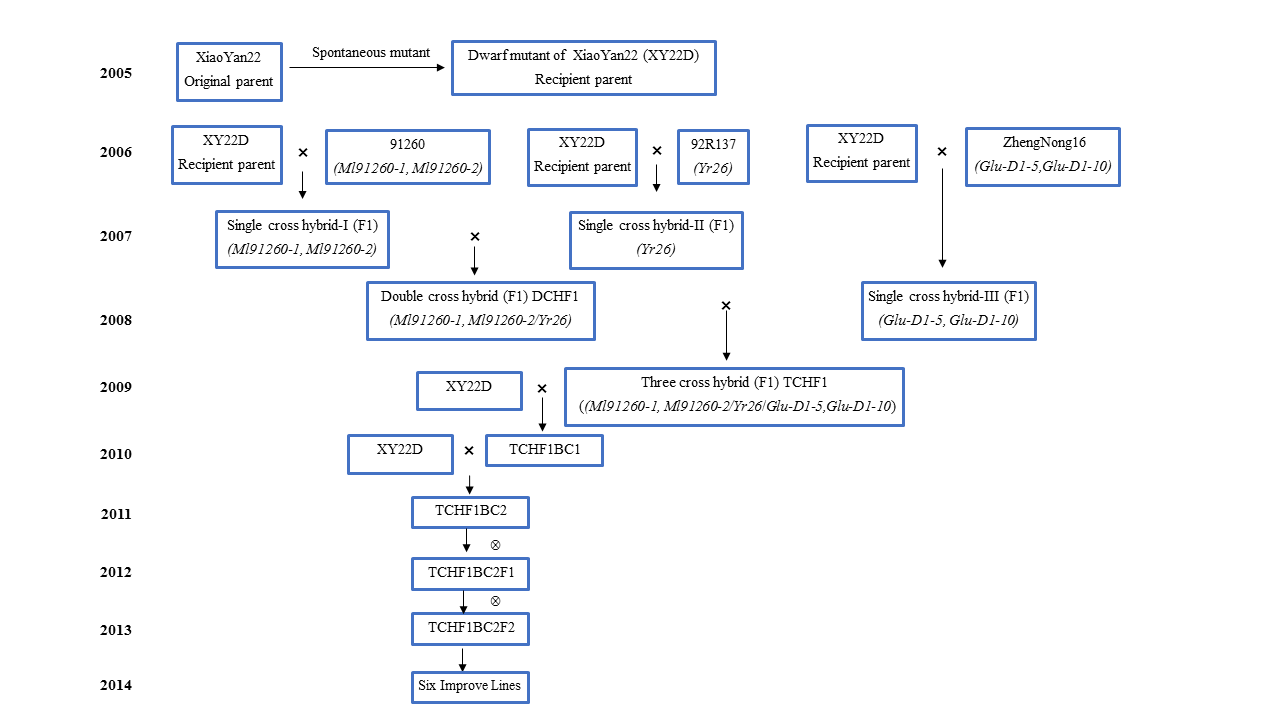

Supplement: Supplementary file 1 — Additional file 1: Figure S1. Flow diagram showing various steps involved in pyramiding of genes in wheat XY22D. [file 12863_2020_854_MOESM1_ESM.tif]
