# Supplementary material for: Molecular marker assisted gene stacking for disease resistance and quality genes in the dwarf mutant of an elite common wheat cultivar Xiaoyan22
Source: BMC Genet. 2020 Apr 23;21:45. doi: 10.1186/s12863-020-00854-2 (PMC7178591; doi:10.1186/s12863-020-00854-2)
Supplement: Supplementary file 2 — Additional file 2: Table S1. Steps involved, and foreground selection exercised for population advancement and pyramiding of genes into the genetic background of wheat XY22D. [file 12863_2020_854_MOESM2_ESM.docx]

| Pyramided Lines/progenies | Number of Plants/Progenies | Traits subjected to foreground selection | Homozygote/ Heterozygote | No. of selected plants |
| --- | --- | --- | --- | --- |
| DCHF1 | 196 | powdery mildew/yellow rust | Heterozygote | 12 |
| TCHF1 | 213 | HWM-GS, powdery mildew/yellow rust | Heterozygote | 15 |
| TCHF1BC1 | 178 | HWM-GS , powdery mildew/yellow rust | Heterozygote | 13 |
| TCHF1BC2 | 143 | HWM-GS , powdery mildew/yellow rust | Heterozygote | 11 |
| TCHF1BC2F1 | 233 | HWM-GS , powdery mildew/yellow rust | Heterozygote | 9 |
| TCHF1BC2F2 | 167 | HWM-GS , powdery mildew/yellow rust | Homozygote | 6 |
